# The Right to Perform Operations in Hospitals

**Published:** 1890-03-15

**Authors:** 


### The Right to Perform Operations in Hospitals.

At an inquest held by Mr. Wynne Baxter, the East London Coroner, on February 22nd, at Shadwell Vestry Hall, on the body of Martha Brett, aged thirteen months, the daughter of a fish porter, residing at 54, Love-lane, Shadwell, the question whether a hospital doctor has a right to perform an operation on a child without the consent of the parents, was raised.

The mother stated that on Wednesday, the 19th inst., the child had a fit of coughing, so she took it to Mr. Brooks, a chemist, of King David Lane, and he advised her to take the child to the Shadwell Children's Hospital. On arrival at the hospital the child was given a warm bath, but as that did not have a beneficial effect the doctor told the mother that an operation to the throat was necessary in order to save life. The mother refused to give her consent to the operation being performed, and left the hospital to consult her husband on the subject. On her return to the hospital she found that the operation had been performed, and the child died shortly afterwards. The mother was very indignant, and stated that the doctor had no right to perform the operation without her consent, adding that if her child had to die she would rather it had died at home than be "murdered" at the hospital.

Mr. Edwin Birchall Hastings (Resident Medical Officer of the Children's Hospital, Shadwell), stated that deceased was admitted to the hospital at 1.40 p.m. suffering from an obstruction in the wind pipe, and was first seen by the house surgeon, Mr. Baker, who gave the child a hot bath. As that did not relieve it Mr. Baker told the mother that the only chance of saving life was to perform trachæotomy. The mother then left the hospital as stated. Meanwhile the condition of the child became so serious that Mr. Hastings' attention was called to it, and as he found it on the point of death, he at once performed trachæotomy, there being no time to wait for the return of the mother. The child was relieved for a short time, but difficult breathing soon returned, and it died about half-an-hour later. The *post mortem* examination showed that death was due to asphyxia caused by a plug of caseous matter in the bronchus. With regard to the charge brought against him of performing the operation without the consent of the parents, Mr. Hastings said that, in his opinion, at the time that was the only chance of saving life, and if he had neglected to operate on the child he considered he should have been gravely neglecting his duty. As a matter of fact, the operation prolonged life for a short time, although it did not save it, but in no way hastened death, which would have taken place without the operation. He submitted to the jury that if he saw a child drowning, and let that child drown, simply because he had not got the parents' consent to save it, he would be, at the least, guilty of gross inhumanity. The coroner went even further than this, and said that he doubted if some juries would not have returned a verdict of manslaughter against the doctor if he neglected to perform an operation because he had not the parents' consent, and the child died for the want of that operation being performed. He considered the doctor was fully justified in acting as he had done; and the jury, also taking this view, returned a verdict in accordance with the doctor's evidence.

This decision on an important point should be borne in mind by all house surgeons; also by all people who, in applying to a hospital for help, practically place themselves in the hands of the authorities.

